# New York Correspondence

**Published:** 1859-08

**Authors:** 


### NEW YORK CORRESPONDENCE.

We published with pleasure, in our last number, Dr. D. C. O'Keefe's letter from New York, and while we are perfectly willing to aid in disseminating a knowledge of the eminent medical advantages of Philadelphia and New York, and have no disposition whatever to detract from the favorable representation given, especially of the *Hospital facilities*, in which they more particularly excel the southern cities, (with perhaps the single exception of New Orleans,) yet, in our judgment, (and in this we are by no means singular,) hospitals are of no very great service to the medical studenr, previous to graduation, and while we think Dr. O'Keefe, ot any other intelligent medical man, who is already prepared to appreciate the practical opportunities afforded—will spend his time more profitably in Philadelphia or New York than in any other city in the United States, save, perhaps, the exception already mentioned (where the hospital advantages are also very extensive), we just as decidedly believe, that medical students can be as well educated in a number of the cities of the south as in those of the north. The fact is, independent of any individual interest we have in medical teaching, we are most uncompromisingly opposed to the continuance of this stream of southern money in the direction of the north.

We acknowledge no inferiority upon the part of the south in any particular, save in a want of development of her resources, the result of a failure upon the part of our own people to foster their own institutions, and use their own advantages, and thus not only depriving themselves of home facilities and independence, but at the same time furnishing the means to those who are in no way identified with their interests, to place themselves in a position to boast of their superior advantages. When will the people of the south awake to their folly? before long, we hope and trust.

We however, commend to the attention of our readers the interesting letter of our intelligent correspondent.

